# Differential effects of N-linked glycosylation of Vstm5 at multiple sites on surface expression and filopodia formation

**DOI:** 10.1371/journal.pone.0181257

**Published:** 2017-07-26

**Authors:** A-Ram Lee, Sulgi Kim, Kwang Woo Ko, Chul-Seung Park

**Affiliations:** 1 School of Life Sciences, Gwangju Institute of Science and Technology (GIST), Gwangju, Republic of Korea; 2 Bioimaging Research Center, Gwangju Institute of Science and Technology (GIST), Gwangju, Republic of Korea; 3 Department of Developmental Biology, Washington University School of Medicine, Saint Louis, Missouri, United States of America; Thomas Jefferson University, UNITED STATES

## Abstract

V-set and transmembrane domain-containing protein 5 (Vstm5), a newly characterized small membrane glycoprotein, can induce membrane protrusions in various cells. Vstm5 can modulate both the position and complexity of central neurons by altering their membrane morphology and dynamics. In this study, we investigated the significance of glycosylation in the expression and function of Vstm5. Four N-linked glycosylation sites (Asn^43^, Asn^87^, Asn^101^, and Asn^108^) are predicted to be located in the extracellular N-terminus of mouse Vstm5. Although all four sites were glycosylated, their functional roles may not be identical. N-glycosylation at multiple sites affects differentially the function of Vstm5. Glycosylation at individual sites not only played essential roles in surface expression of Vstm5 but also in the formation of neuronal dendritic filopodia. These results indicate that N-linked glycosylation at multiple sites plays important roles by differentially influencing the expression, targeting, and biological activity of Vstm5.

## Introduction

V-set and transmembrane domain-containing protein 5 (Vstm5) is a small putative cell-adhesion molecule belonging to the immunoglobulin superfamily abundantly expressed in mouse brain. Vstm5 is highly expressed at postnatal day 1 in mouse brain, when synaptogenesis actively occurs [1]. This temporal expression pattern suggests that Vstm5 modulates neuronal connectivity and plasticity. In fact, Vstm5 regulates neuronal morphology by redistributing F-actin and promotes synapse formation [2]. In addition, Vstm5 promotes neurite formation in the leading processes of migrating neurons to facilitate dendrite formation and integration of neurons into the appropriate laminae of the developing mouse cortex [2].

Our previous study showed that Vstm5 can be glycosylated at multiple sites and suggested that four potential N-linked glycosylation sites are located in its extracellular domain [2]. Cellular N-glycosylation is highly regulated in response to developmental, physiological, and environmental cues, in part through changes in the expression of glycosyltransferases that function in the endoplasmic reticulum (ER) and the Golgi [3]. In many systems, glycosylation plays a role in surface expression as well as protein stability [4, 5]. For example, several membrane proteins, including ion channels, transporters, and receptors that contain N-linked glycan in their mature forms, are involved in membrane targeting [6–11]. Recent reports indicate that impaired N-glycosylation can cause various disorders of the central nervous system [12]. For example, abnormal N-glycosylation of neurotransmitter-related proteins and amyloid precursor protein (APP) has been reported in patients with schizophrenia and Alzheimer disease (AD) [13–16].

In this study, we characterized the effect of N-linked glycosylation of Vstm5 on its expression and function. We showed that all four N-linked glycosylation sites predicted by bioinformatic analysis are in fact glycosylated in the cell. However, glycosylation at each site had differential effects on the surface expression of Vstm5 and filopodia induction. Thus, N-linked glycosylation at multiple sites appears to be critical for the expression, targeting, and function of Vstm5.

## Materials and methods

### DNA constructs and molecular cloning

The full-length complementary DNA (cDNA) of the Vstm5 protein was purchased from CloneRanger (Invitrogen). Vstm5 cDNA was amplified using PCR and then subcloned in-frame into pEGFP-N3 (Clonetech). The C-terminus of Vstm5 was also tagged with a monoclonal antibody epitope (Vstm5::1D4) by replacing EGFP in pEGFP-N3 with a C-terminal rhodopsin 1D4 tag [17]. To construct the C-terminal point mutants, Vstm5-N43A, -N87A, -N101A, -N108A, -N43,87A, -N101,108A, -N87,101,108A, and -N43,87,101,108A were subcloned into pEGFP-N3 tagged with the green fluorescent protein (GFP) or 1D4 epitope.

### Antibodies

The following antibodies commercially available were used: a rabbit polyclonal antibody directed against recombinant full length GFP (1/1000; ab290, Abcam); a mouse monoclonal [1D4] directed against cow rhodopsin (1/1000; ab5417, Abcam); and a rabbit polyclonal directed against microtubule-associated protein 2 (1/500; sc-20172, Santa Cruz Biotechnology). The secondary antibody used for immunocytochemistry was an Alexa Fluor-conjugated antibody (Alexa 488, Alexa 594, and Alexa 633; 1/1000; Invitrogen). For western blot analysis, a horseradish peroxidase-conjugated secondary antibody (1/10000; Jackson ImmunoResearch Laboratories) was used. Texas Red-X Phalloidin was used to assess filamentous actin (1/1000; T7471, Molecular Probes).

### Cell culture and transfection

Both human embryonic kidney 293 cells (HEK 293) and African green monkey kidney fibroblast-like cells (COS-7) were cultured at 37°C in Dulbecco’s Modified Eagle’s Medium (Hyclone) supplemented with 10% fetal bovine serum (Hyclone) in a humidified atmosphere containing 5% CO_2_. Cells were transfected using Lipofectamine 2000 (Invitrogen) according to the manufacturer’s instructions. Wild-type ICR mice with 18 days of pregnancy were purchased from Damul Science (Damul Science, Daejeon, Korea). All experiments used protocols approved by the Animal Care and Ethics Committees of the Gwangju Institute of Science and Technology (permit number: GIST-2014-52) in accordance with the National Institutes of Health Guide for the Care and Use of Laboratory Animals. Mouse hippocampal neuronal cultures were prepared as described previously [18]. Briefly, hippocampi were dissected from E18 mice and dissociated with papain (Worthington Biochemical Corp). The cells were plated on poly-D-lysine-coated cover glasses at a density of 5 × 10^5^ cells per 60-mm plastic dish. They were maintained in Neurobasal medium (Gibco) supplemented with B-27 (Invitrogen) and 2 mM L-GlutaMAX (Invitrogen). Neurons were transfected at day *in vitro* (DIV) 1 or 2 using a modified calcium-phosphate precipitation method [19].

### Deglycosylation of Vstm5 using tunicamycin and enzymes

Cells were transiently transfected with Vstm5 constructs. Six hours after transfection, tunicamycin was added to the medium at various concentrations (0, 0.5, 1.0, and 5.0 μg/ml). Cells were harvested 4 h after the addition of tunicamycin using RIPA buffer (20 mM HEPES, 150 mM NaCl, 1 mM EDTA, 1 mM EGTA, 1% sodium deoxycholic acid, 1% Triton X-100, 2 mM Na_3_VO_4_, 1% NP-40, 2 mM NaF, and 1× protease inhibitor cocktail [Complete; Roche Diagnostics]). In order to remove glycosylation of Vstm5, the cells were treated with endoglycosidase H (endo H) or peptide-N-glycosidase F (PNGase F) (New England Biolabs) at 1U/μg protein for 16 h at 37°C, according to the manufacturer’s instructions. The effects of chemical and enzymatic treatments on Vstm5 glycosylation were analyzed using SDS-PAGE, followed by immunoblotting. Bands corresponding to surface biotinylated WT and mutant Vstm5 were quantified using ImageJ software and then normalized to the amount of total lysate.

### Biotinylation and western blot analysis

Transfected COS-7 cells were rinsed twice in phosphate-buffered saline (PBS^2+^) supplemented with 0.1 mM CaCl_2_ and 1 mM MgCl_2_ and incubated with Sulfo-NHS-LC-biotin (Thermo Scientific) at a concentration of 0.5 mg/ml for 1 h on ice. The cells were rinsed three times in PBS^2+^ and lysed in 1 ml of lysis buffer (25 mM Tris-HCl [pH 7.5], 150 mM NaCl, 1% sodium deoxycholic acid, 1% Triton X-100, 0.1% SDS, 1 mM phenylmethylsulfonyl fluoride, and 1× protease inhibitor cocktail [Complete; Roche Diagnostics]) for 30 min on ice. Aliquots of the lysates (10% of the total cell lysate) were subjected to western blot analysis with an anti-1D4 antibody. Biotinylated proteins were precipitated from the remaining 90% of the lysate with streptavidin-agarose beads (Thermo Scientific). Precipitates were washed three times with PBS and eluted into sample buffer. Samples were resolved by SDS/PAGE and immunoblotted with an anti-1D4 antibody at 4°C overnight. After extensive washing in 1 × TBST, the membrane was incubated with a horseradish peroxidase-conjugated secondary antibody (Jackson ImmunoResearch Laboratories). Proteins were visualized with ECL reagent (GE Healthcare Life Sciences).

### Immunofluorescence and confocal microscopy

COS-7 cells and hippocampal neurons were washed three times with PBS and then with 4% paraformaldehyde/sucrose prepared in PBS for 15 min at room temperature. After washing, the cells were permeabilized with 0.25% Triton X-100 prepared in PBS for 5 min at room temperature and blocked with 5% bovine serum albumin prepared in PBS for 30 min. The cells were incubated with a primary antibody for 1 h and subsequently with a fluorescence-conjugated secondary antibody for 45 min. Fluorescence images were acquired using a Fluoview FV 1000 confocal laser-scanning microscope equipped with 100× oil-immersion objectives and capable of an additional ×4 zoom.

### Image analysis and quantification

Analysis and quantification of data were performed using ImageJ. In COS-7 cells, the total number of filopodia was counted, and the total number was divided by the length of cell circumference to generate the number of filopodia per μm. In neurons, we imaged at 7 DIV when dendritic spine induction has not yet occurred, and filopodia are the predominant dendritic protrusion. We thus defined dendritic filopodia as any protrusion under 10 μm in dendritic length. Statistical comparisons were performed by ANOVA followed by a Dunnett’s posthoc test making comparison against a single control group. P values of less than 0.05 were considered significant. Data are presented as mean ± standard error of the mean (SEM).

## Results

### Vstm5 is a glycoprotein with multiple N-linked glycosylation sites

**Fig 1:**
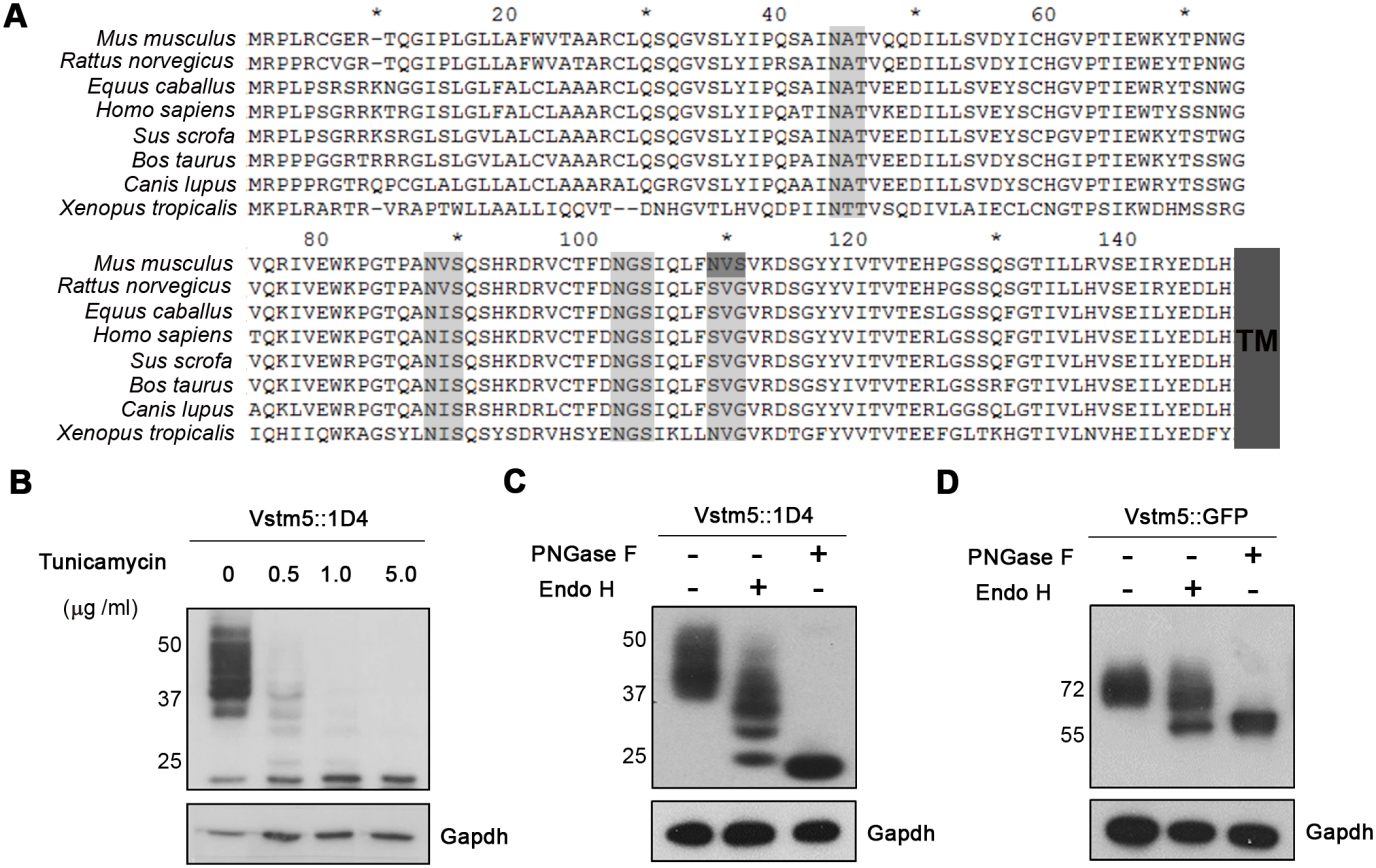
Locations of putative N-glycosylation sites and confirmation of N-glycosylation of Vstm5. **(A)** The extracellular region sequences of Vstm5 from *Mus musculus* (NP_081231), *Rattus norvegicus* (NP_001138342), *Equus caballus* (XP_001491444.3), *Homo sapiens* (NP_001138343), *Sus scrofa* (XP_003129835), *Bos taurus* (NP_001193893), *Canis lupus* (XP_005633416), and *Xenopus tropicalis* (XP_004912166) were multi-aligned using Clustal X. The conserved N-linked glycosylation sites and transmembrane domain are boxed in light gray and dark gray, respectively. **(B)** COS-7 cells transfected with Vstm5::1D4 were treated with tunicamycin at the indicated concentration for 6 h after transfection. **(C, D)** Total Vstm5::1D4-expressing (C) or Vstm5::GFP-expressing (D) cell lysates were treated with 1U endoH and then 1U PNGase F. Samples were resolved by SDS-PAGE, followed by western blotting with an anti-1D4 or anti-GFP antibody. Gapdh served as loading control.

Vstm5 is a highly conserved protein in different species, especially in mammals [2]. While three out of four (Asn^43^, Asn^87^, and Asn^101^) predicted N-linked glycosylation sites are conserved in the extracellular domain, one site (Asn^108^) is found specifically in mouse (Fig 1A). This Asn residue is replaced by Ser residues in other species, except *Xenopus*. As we previously reported [2], Vstm5 was detected as multiple protein bands at apparent molecular weights of 30–45 kDa. Treatment with tunicamycin, an antibiotic that blocks the synthesis of N-linked oligosaccharide chains on glycoproteins, shifted the multiple protein bands to a single band of approximately 20 kDa in a dose-dependent manner (Fig 1B). To determine the glycan maturation of Vstm5 in COS-7 cells at steady state, cell lysates expressing Vstm5::1D4 or Vstm5::GFP were digested with endo H or PNGase F to remove the entire glycan chain (Fig 1C and 1D). Digestion with endo H generated protein bands of around 20–30 kDa for Vstm5::1D4 (Fig 1C) or 55kDa for Vstm5::GFP (Fig 1D), respectively, termed endo H-sensitive bands. Because endo H cleaves immature proteins, the endo H-sensitive bands represent Vstm5 that has not been processed beyond the endoplasmic reticulum (ER). Digestion with PNGase F also generated a protein band at 21 kDa (Fig 1C) or 55kDa (Fig 1D), representing fully deglycosylated Vstm5. These results indicate that Vstm5 is glycosylated mostly, if not absolutely, by N-linked forms and that the majority of Vstm5 is expressed as an endo H-sensitive mature form in cells.

### N-glycosylation is critical for surface expression of Vstm5

**Fig 2:**
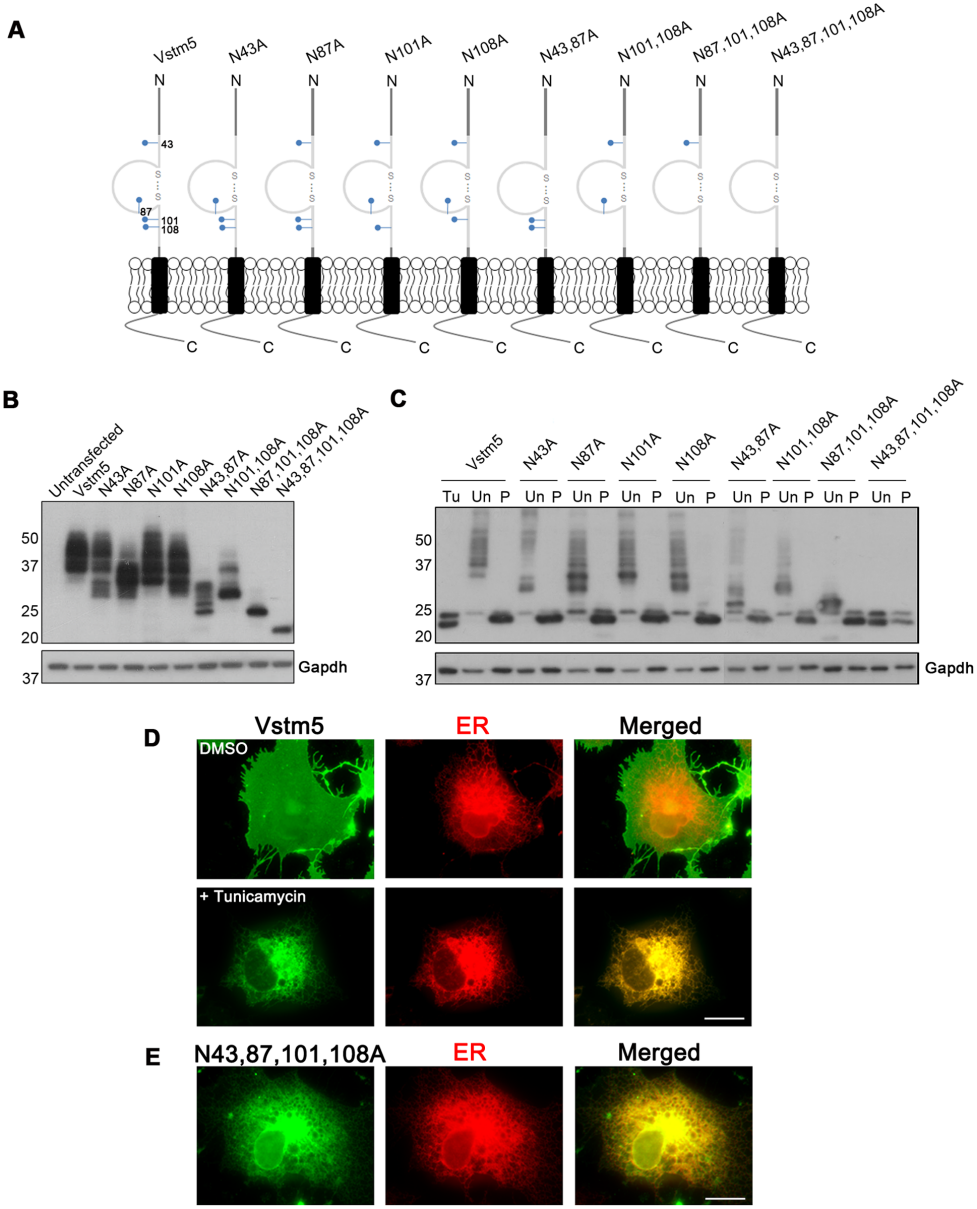
N-linked glycosylation of Vstm5 at multiple sites. **(A)** A schematic illustration of Vstm5 mutants harboring mutations in the four putative N-glycosylation sites. Blue lines with filled circles indicate glycosylation sites (Asn^43^, Asn^87^, Asn^101^, and Asn^108^). **(B)** Each mutant was transfected into COS-7 cells. **(C)** Lysates of COS-7 cells expressing WT and mutant Vstm5 were treated with tunicamicin or PNGase F, followed by SDS-PAGE and western blotting with an anti-1D4 antibody. Tu, tunicamycin-treated. Un, untreated control. P, PNGase F-treated. Gapdh served as loading control. **(D)** Vstm5-transfected COS-7 cells were treated with DMSO (control) or 1 μg/ml tunicamycin for 24 h and immunostained with an anti-1D4 antibody (green) and an anti-PDI antibody (red). (E) COS-7 cells transiently transfected with the Vstm5 (N43,87,101,108A) mutant were immunostained with an anti-1D4 antibody (green) and an anti-PDI antibody (red). Scale bar, magnification: 30 μm.

To confirm the glycosylation of the four candidate Asn residues, we generated 1D4-tagged Vstm5 mutants. The four Asn residues were replaced by Ala individually or in combination, and the mutants were expressed in COS-7 cells (Fig 2). All four single mutations changed the migration patterns of the protein bands and shifted them to positions lower than that of wild-type (WT) Vstm5 (Fig 2B and 2C), indicating that all four predicted sites (Fig 2A) were individually glycosylated in the cell. Most single and double mutants were detected at around 20–30 kDa and also at multiple bands around 37 kDa (fully matured form). These results indicate that the glycosylation of multiple sites is critical for proper trafficking of Vstm5 from the ER to the Golgi apparatus or from the Golgi apparatus to the plasma membrane. By contrast, the triple and quadruple mutants were detected only at around 21 kDa, indicating that they are trapped in the ER or retained in other compartments after exiting the ER.

Vstm5 was glycosylated at multiple sites; therefore, their functional importance was further investigated. We wondered whether the inhibition of glycosylation interferes with the targeting of Vstm5 to the cell membrane and/or the formation of membrane protrusions. To determine its subcellular distribution, the expression pattern of Vstm5 was compared with that of an ER marker, protein disulfide isomerase (PDI). WT Vstm5 was mainly expressed at the membrane edges of COS-7 cells and induced membrane protrusions (Fig 2D). In the presence of tunicamycin, however, Vstm5 was trapped at the perinuclear region and co-localized with PDI. These results strongly suggested that N-linked glycosylation of Vstm5 was necessary for its proper targeting to the plasma membrane. Consistently, the quadruple mutant (N43,87,101,108A) had a similar intracellular localization pattern and failed to induce membrane protrusions (Fig 2E). Thus, N-linked glycosylation of the extracellular domain may be critical for the surface expression of Vstm5.

### N-linked glycosylation has differential effects on membrane expression of Vstm5

**Fig 3:**
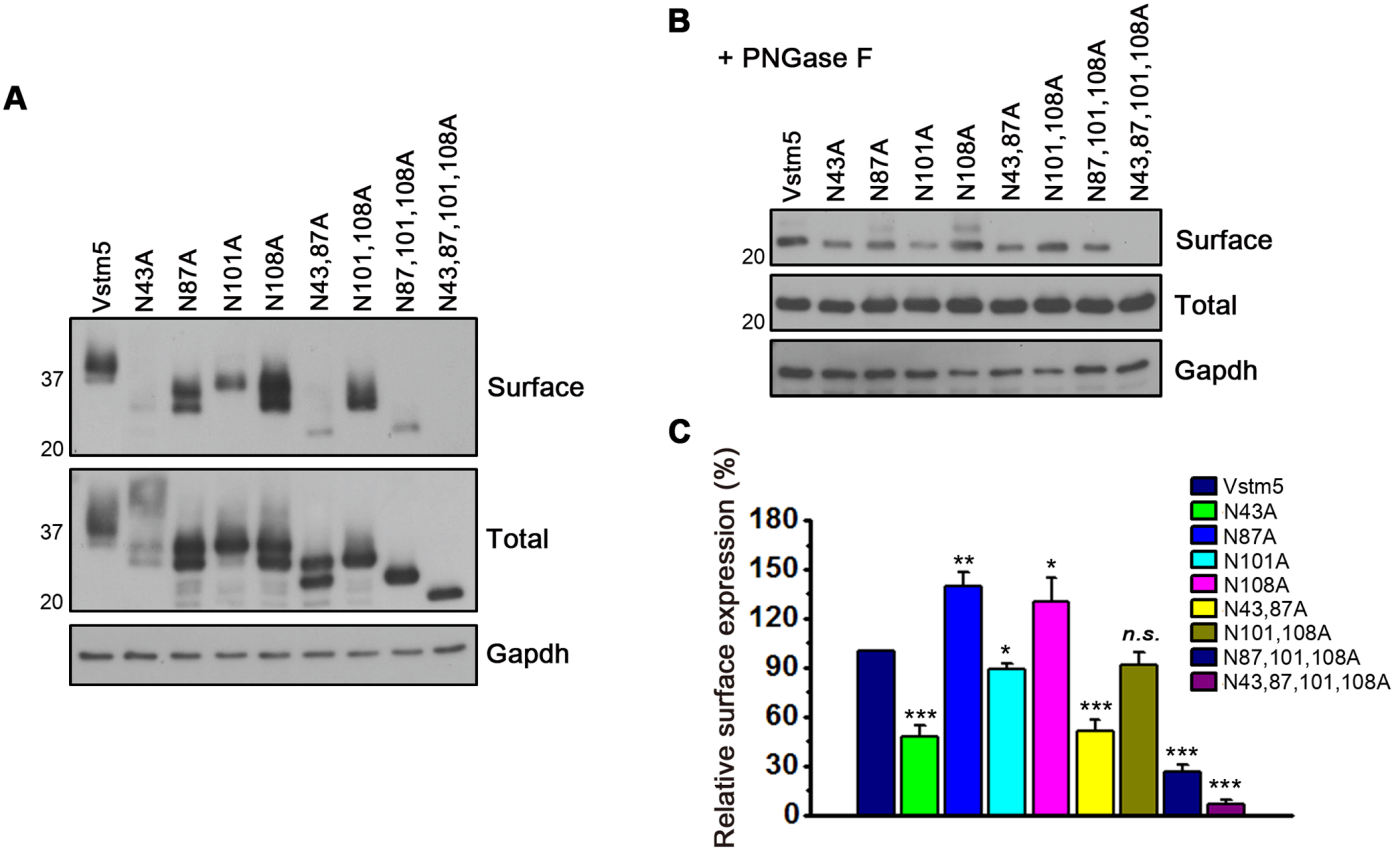
Plasma membrane expression of WT and mutant Vstm5. **(A, B)** WT Vstm5 and its glycosylation mutants expressed in cells were biotinylated and subsequently precipitated using Neutravidin-agarose. The Vstm5-containing lysates were treated without (A) or with PNGase F (B). **(C)** Surface levels of biotinylated WT and mutant Vstm5 were normalized to that of total protein in (B). The western blot is representative of three individual experiments. Data represent means ± SEM (*P<0.05, **P<0.01, ***P<0.001, *n*.*s*., no statistical significance, significantly different from WT Vstm5 by an ANOVA and Tukey’s HSD post hoc test).

N-linked protein glycosylation is crucial for the trafficking of several membrane proteins toward the plasma membrane [4, 20, 21]. To appreciate the effects of N-linked glycosylation of individual sites on surface expression, we performed a surface biotinylation assay using different glycosylation mutants (Fig 3A). In order to measure the intensity of total protein bands accurately, PNGase F was added to each lysate to remove glycosylation before performing the pull-down procedure. The western bands shown in Fig 3B were quantified for comparison. Among the single point mutants, the plasma membrane expression of N43A and N101A was lower than that of WT Vstm5. Intriguingly, the surface expression of N87A and N108A was increased (1.4-fold and 1.3-fold, respectively) compared with that of WT Vstm5. Among the multiple point mutants, surface expression was significantly decreased for all mutants except for the N101,108A double mutant. The level of N101,108A in the plasma membrane was not significantly different with that of WT Vstm5 (Fig 3C). These results demonstrate that N-linked glycosylation at Asn^43^ sites was critical for surface expression of Vstm5. However, glycosylation at Asn^87^and Asn^108^ was not necessary for efficient recruitment of Vstm5 to the plasma membrane.

### N-linked glycosylation modulates filopodia induction by Vstm5

**Fig 4:**
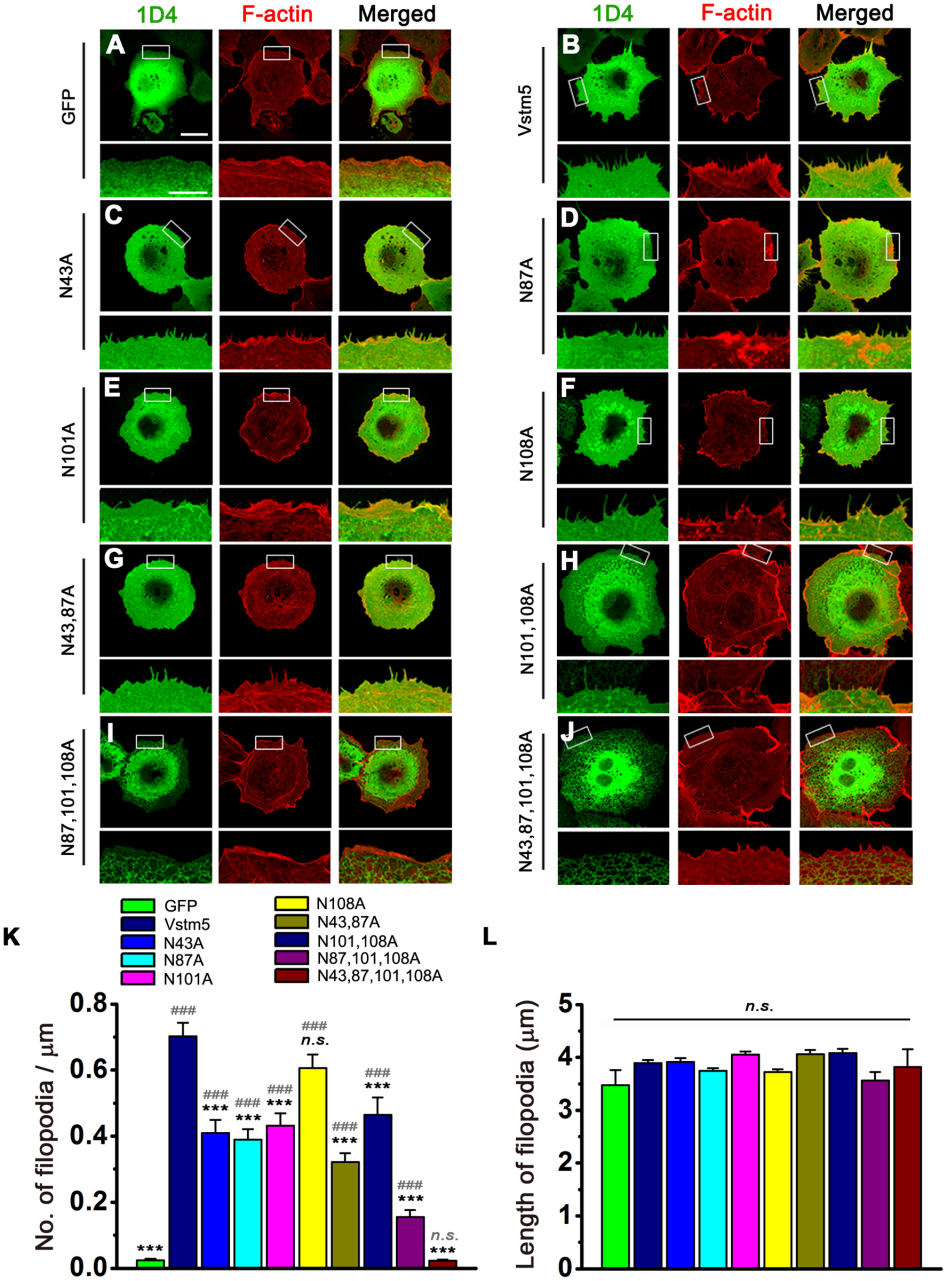
Effects of N-glycosylation on Vstm5-induced filopodia formation. **(A–J)** Immunocytochemistry of WT Vstm5::1D4 and its glycosylation mutants. Transfected COS-7 cells were stained with an anti-1D4 antibody (green), and phalloidin (red). Higher magnification views of the regions enclosed by the rectangle are shown below each image. Scale bar, low magnification: 30 μm; high magnification: 10 μm. **(K, L)** Histograms of the number (K) and length (L) of filopodia were quantified and divided by the length of cell circumference. Analysis was performed using Image J (public domain, Wayne Rasband, USA). Data represent means ± SEM (GFP, n = 25; Vstm5, n = 64; Vstm5 [N43A], n = 61; Vstm5 [N87A], n = 57; Vstm5 [N101A], n = 66; Vstm5 [N108A], n = 67; Vstm5 [N43,87A], n = 68; Vstm5 [N101,108A], n = 38; Vstm5 [N87,101,108A], n = 30; Vstm5 [N43,87,101,108A], n = 28). *, indicates significant difference compared with WT Vstm5, *P<0.05, **P<0.01, ***P<0.001. #, indicates significantly different from GFP versus Vstm5 or mutants, ^#^*P*<0.05, ^##^*P*<0.01, ^###^*P*<0.001. *n*.*s*., no statistical significance.

Since the conventional filopodial structures are tightly linked to parallel bundles of actin filaments [22, 23], F-actin can be visualized by labeling the cells with phalloidin in filopodial structure. We reported previously that Vstm5 induces filopdoia and colocalizes with F-actin in neuronal and non-neuronal cells [2]. To verify the effects of N-glycosylation on filopodia induction, we analyzed the number and length of filopodia in COS-7 cells. WT and mutant Vstm5 were individually tagged with 1D4 and transfected into COS-7 cells, and then cells were stained with an anti-1D4 antibody (green) and phalloidin (red) (Fig 4A). The density of filopodia was higher in cells expressing Vstm5::1D4 (Fig 4B) than in GFP-transfected control cells (Fig 4A and 4K). In cells expressing the N43A, N87A, and N101A single mutants, the density of filopodia was significantly reduced compared with that of WT Vstm5 (Fig 4B, 4C, 4D, 4E and 4K). However, the N108A mutant induced filopodia as potently as WT Vstm5, suggesting the functional insignificance of Asn^108^ in filopodia induction (Fig 4F and 4K). Effects of multiple mutations on the ability of Vstm5 to induce membrane protrusion in COS-7 cells were also complex and somewhat additional. The number of membrane protrusions induced by a triple mutant, N87,101,108A, was greatly reduced compared with that of WT Vstm5 or with the individual single mutants. The quadruple mutant N43,87,101,108A failed to target the cell surface and did not colocalize with F-actin (Fig 4J). As expected, the quadruple mutant could not induces additional membrane protrusion compared with GFP control (Fig 4K). Despite the marked effects of WT and mutant Vstm5 on the filopodial density, the length of filopodia was not significantly affected (Fig 4L). These results indicate that N-glycosylation of Vstm5 is closely related not only to its surface expression, but also to filopodia induction.

### N-linked glycosylation modulates induction of neuronal dendrites by Vstm5

**Fig 5:**
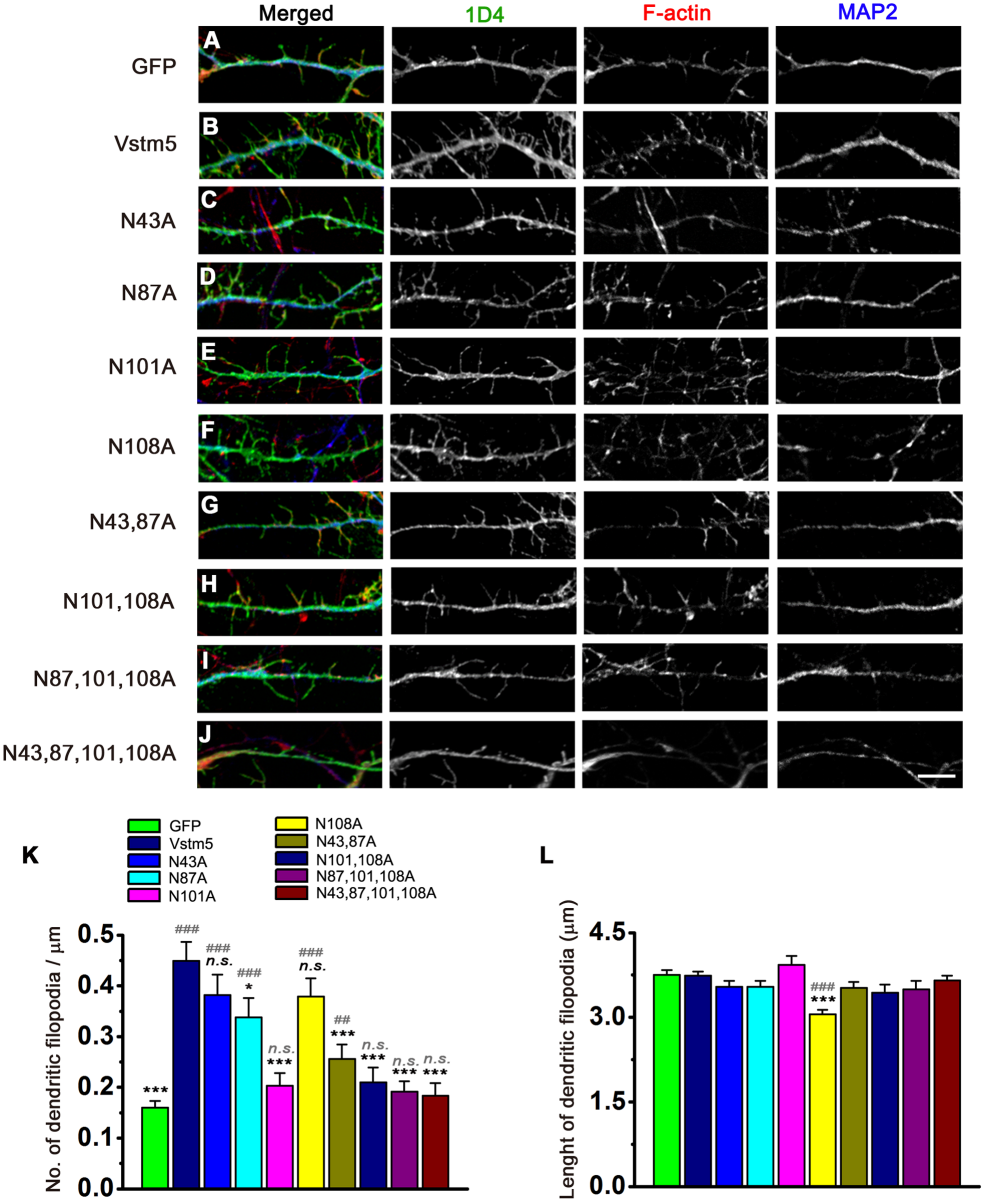
Effects of N-linked glycosylation on Vstm5-induced neuronal filopodia. **(A–J)** Mouse hippocampal neurons (DIV 7) were transfected with control GFP, WT Vstm5::1D4, or N-linked glycosylation mutants. Immunocytochemistry was performed using an anti-1D4 antibody (green), phalloidin (red), and an anti-MAP2 antibody (blue). Scale bar: high magnification, 5 μm. **(K, L)** The number (K) and length (L) of dendritic filopodia. Data represent means ± SEM (GFP, n = 46; Vstm5, n = 71; Vstm5 [N43A], n = 58; Vstm5 [N87A], n = 49; Vstm5 [N101A], n = 46; Vstm5 [N108A], n = 66; Vstm5 [N43,87A], n = 50; Vstm5 [N101,108A], n = 33; Vstm5 [N87,101,108A], n = 33; Vstm5 [N43,87,101,108A], n = 14). *P<0.05, ***P<0.001 compared to the WT Vstm5; ^#^*P*<0.05, ^##^*P*<0.01, ^###^*P*<0.001 compared to the GFP; *n*.*s*., no significance.

In early synaptogenesis, dendritic filopodia play critical roles in actively initiating contacts with nearby axonal shafts, and pre- and postsynaptic molecules are rearranged and thereafter evolve into dendritic spines [24]. In our previous study [2], we found that Vstm5 induces mainly filopodia on the dendritic regions of a neuron. To examine the functional effects of N-linked glycosylation on Vstm5-induced formation of dendritic filopodia, we transfected WT Vstm5 and N-linked glycosylation mutants of Vstm5 into mouse hippocampal neurons (DIV 1). The neurons were stained for F-actin with phalloidin and for MAP2, a dendritic marker, with anti-MAP2 antibody. As previously reported [2], the number of dendritic filopodia was higher in WT Vstm5-expressing neurons than in GFP-expressing control neurons (Fig 5A, 5B and 5K). However, the single mutations affected differentially Vstm5 for its ability to induce dendritic filopodia. While the single N-glycosylation mutants N87A and N101A exhibited significantly fewer dendritic filopodia than those transfected with WT Vstm5 (Fig 5B, 5D, 5E and 5K), both N43A and N108A showed no significant differences in the number of dendritic filopodia compared with WT Vstm5 (Fig 5B, 5C, 5F and 5K). The reducing effects of dendritic filopodia was most prominent for N101A (Fig 5E and 5K), suggesting the importance of N-glycosylation on this position. We then tested the multiple mutants for their ability to induce dendritic filopodia, since these mutant Vstm5 (N43,87A, N101,108A, and N87,101,108A) were targeted to cell surface and able to induce some membrane protrusions in COS-7 cells (Fig 4G, 4H and 4I). Surprisingly, however, the multiple mutants only except N43,87A was not able to increase the number of filopodia significantly in the dendritic regions of transfected neurons compared with the GFP-transfected control neurons (Fig 5G, 5H and 5I). As expected in COS-7 cells, the quadruple mutant could not induces dendritic filopodia same as GFP control (Fig 5K). All of the Asn^101^ loss mutants are decreased in dendritic filopodia formation. These results indicate that N-glycosylation at Asn^101^ is the most important modification for dendritic filopodia formation.

The length of dendritic filopodia in neuron transfected with WT Vstm5 and any of the mutants were not significantly different, except N108A in which the dendritic filopodial length was significantly shorter than rest of them (Fig 5L). These results are partially consistent with the aforementioned findings in COS-7 cell lines and further indicate the functional significance of glycosylation of the extracellular domain of Vstm5 in the formation of neuronal dendritic filopodia.

## Discussion

Among protein post-translational modifications, the most well-studied and most complex modification is the N-linked glycosylation of the Asn residue in the consensus motif Asn-X-Ser/Thr [25]. N-glycosylation, which mainly occurs in secreted, cytosolic or membrane proteins, plays important roles in correct protein folding, assembly and stability [3], as well as in cell adhesion and diverse signaling pathways [26, 27]. The emergence of a new N-glycosylation site during evolution can alter protein function either positively or negatively [28]. In the present study, we showed that Vstm5, a newly characterized membrane protein, undergoes N-linked glycosylation at multiple sites. This glycosylation is critical not only for the expression of Vstm5 at the plasma membrane but also for the induction of membrane protrusions. Treatment with tunicamycin, which inhibits the action of glycosyltransferases in the ER, and mutation of the four predicted N-linked glycosylation sites prevented Vstm5 from reaching the cell surface and caused it to become trapped in the ER (Fig 2D and 2E). These results suggested that glycosylation is a key element for the proper trafficking and surface expression of Vstm5. In general, the ER has been recognized as a checkpoint for protein quality control [29]. The localization of non-glycosylated forms of Vstm5 in the ER suggests that adequate glycosylation is required for Vstm5 to transit to the Golgi complex for further processing. The decreased stability of other membrane proteins has been attributed to a lack of glycosylation [5, 30], also raising the possibility that immature glycosylation may direct Vstm5 for degradation.

Three Asn residues in the N-terminal extracellular domain of Vstm5 (Asn^43^, Asn^87^, and Asn^101^) are well conserved in vertebrates, implying that N-linked glycosylation is a common post-translational modification of vertebral Vstm5. We found that N-glycosylation can modulate the surface expression of Vstm5 and the formation of filopodia-like structures. It was reported that some proteins, such as the ß4 subunit of sodium channel and the acid-sensing ion channel-1a (ASIC1a), can induce the filopodia-like structures in a glycosylation-dependent manner [9, 10]. Especially, N-glycosylation at Asn^393^ and Asn^366^ in ASIC1a has differential effects on ASIC1a biogenesis. The N366Q mutant of ASIC1a, which shows increased surface expression, glycosylation and dendritic targeting, potentiated acidosis-induced spine loss. Conversely, the N393Q mutant of ASIC1a, which showed a decrease in dendritic targeting and inhibition of ASIC1a current dominant-negatively, had the opposite effect [9].

Mutation of four N-linked glycosylation sites in Vstm5 had significant functional effects on its expression and filopodia induction. Although total protein expression was not significantly affected by glycosylation mutations, targeting to the cell surface was differentially affected by these mutations (Fig 3). While N43A and N101A has lower surface expression (Fig 3), only the N101A mutant was important for filopodia formation (Fig 5). These results indicate that the Asn^101^ residue is not only critical for the recruitment of Vstm5 to the plasma membrane but is also essential for the formation of dendritic filopodia in neurons. The cell surface expression of the N87A and N108A was even higher than that of WT Vstm5 (Fig 3). Despite its ample expression at the cell surface, this mutant failed to induce more membrane filopodia than WT Vstm5 (Figs 4K and 5K), indicating that surface expression *per se* is not sufficient for the formation of membrane protrusions. Therefore, four different N-glycosylation sites in Vstm5 have differential effects on the formation of filopodia as well as the surface expression.

It is worth noting that the N108A mutant successfully expressed on the cell surface and induces significantly shorter filopodial extensions than WT Vstm5 and GFP control (Fig 5L). This result suggests that glycosylation of the Asn^108^ residue may be important for dendritic spine maturation of neurons. The Asn^108^ residue is an N-linked glycosylation site found specifically in mouse Vstm5 compared to other mammals (Fig 1A). Thus, glycosylation at Vstm5-Asn^108^ may play other functional roles specific to mouse. During evolution, the gain of new N-glycosylation sites is known to affect the protein structural and molecular function [28]. The new glycosylation sites can be preserved during evolution, when the novel modifications confer beneficial properties.

The morphology of neurons is important for the functional neural circuit and is related to brain pathophysiology. As previously reported, Vstm5 increased dendritic complexity and synapse formation *in vivo* as well as *in vitro* and controlled neuronal migration in the brain [2]. N-linked glycosylation can also affect neuronal morphology and synapse formation during neuronal development [31]. Many studies have reported that abnormal N-glycosylation of membrane proteins are associated with various brain diseases including schizophrenia and depression [13–15, 32]. Thus, it will be of interest to determine whether the Vstm5-Asn^101^ (the key residue of Vstm5, inducing filopdoia) and Vstm5-Asn^108^ (the evolutionary adapted residue, inducing short dendritic filopodia) have any functional consequences for the development and maturation of the neuronal circuit and brain function in general. It is also worth noting that Vstm5 was recently identified as one of the target genes responsible for the differential response to the treatment of major depressive disorder [33], implying possible roles of Vstm5 in psychiatric diseases.

Taken together, our results strongly suggest that N-glycosylation at multiple sites not only plays essential roles in surface expression of Vstm5 but also the formation of neuronal dendritic filopodia. Moreover, glycosylation at individual sites may differentially affect the function of Vstm5 by influencing the expression and maturation of this small membrane protein with a simple structure.
